# Development of a self-report measure of GPS uses and its relationship with environmental knowledge and self-efficacy and pleasure in exploring

**DOI:** 10.1186/s41235-024-00605-2

**Published:** 2024-11-28

**Authors:** L. Miola, V. Muffato, A. Boldrini, F. Pazzaglia, C. Meneghetti

**Affiliations:** 1 Department of General Psychology, University of Padova, Via Venezia, 8, 35131 Padua, Italy; 2 Interuniversity Research Center in Environmental Psychology (CIRPA), University of Rome “La Sapienza”, Via Dei Marsi 78, 00185 Rome, Italy

## Abstract

The widely utilized Global Positioning System (GPS) plays a crucial role in everyday navigation. The literature has predominantly focused on GPS use for reaching destinations rather than exploring its various strategic applications and relations with individual factors. The current paper is intended to develop a GPS Uses Scale assessing a variety of GPS uses for wayfinding and other GPS uses (Study 1). We also examine whether GPS uses are related to gender, age, self-efficacy and pleasure in exploring, dependence on GPS devices, and environment knowledge (Study 2). In Study 1, 365 participants completed the new GPS Uses Scale and the McGill GPS questionnaire, for assessing validity. Results from the confirmatory factor analysis confirmed a structure as five-level factors, good reliability, and validity. In Study 2, 200 participants completed the GPS Uses Scale, self-efficacy and pleasure in exploring scale, GPS dependence scale, and a sketch map task after learning a virtual city from a video. Results from the linear model showed that those who use GPS for strategic purposes reported higher self-efficacy and pleasure in exploring as well as dependence on GPS. Moreover, those who use GPS for orientation purposes reported higher dependency on GPS and had higher scores on the map task (environment knowledge). Men were less likely to use GPS for orientation. The present paper outlines the importance of assessing the various uses of GPS, suggesting self-efficacy and dependence on GPS, and contributes to its strategic use.

## Introduction

The ability to navigate and orient plays a crucial role in our everyday lives; we use this ability to maintain our sense of direction and location while traveling, to familiarize ourselves with new surroundings, and to plan routes to destinations in places we do not know (or those we know well; Montello, 2001, 2005).

Nowadays, navigation and orientation should be considered in relation to technological progress, particularly with the advent of digital navigation aids that make use of Global Positioning System (GPS) (Dalton et al., 2019).

GPS consists of satellite signals that provide accurate coordinates to users, becoming essential for both pedestrian and automotive navigation. Most of us have experience using GPS, even daily, for ordinary movements in both familiar and unfamiliar environments. The use of this technological tool inevitably interacts with orientation skills, and there is a need to better understand this relationship (Ishikawa, 2019). The most widely used instrument to measure GPS use is the McGill GPS questionnaire, which is divided into two subscales—the GPS dependence and reliance scales (Dahmani & Bohbot, 2020). The reliance scale rates the frequency of using GPS in many situations like traveling new routes in unfamiliar environments, or previous visited destinations, and familiar route. The dependence scale instead assesses whether people feel anxious when driving without GPS, tend to get lost without GPS, and feel comfortable in using GPS. However, the literature lacks measures of other GPS usage than simply reaching a destination or how dependent one feels on it, such as understand own location, explore unfamiliar areas, plan travel times, and other practical applications in individuals’ daily routines. Therefore, the present paper sought to advance research in the field by developing a self-report tool to assess the various GPS uses, highlighting a range of behaviors and strategies. Our objective was to move beyond traditional GPS use for wayfinding and reaching a destination and explore less typical or alternative uses for other everyday purposes (also related to space and orientation).

*GPS use and navigation abilities* From a cognitive point of view, navigation ability is a multidimensional construct (Wiener et al., 2009; Wolbers & Hegarty, 2010), combining a wayfinding ability that delves into planning, decision-making, and knowledge of the environment regarding paths, locations, landmarks, and metric configuration of the environment (Dalton et al., 2019; Wiener et al., 2009; Wolbers & Hegarty, 2010).

Studies have highlighted significant differences in navigation abilities among individuals (e.g., Ishikawa & Montello, 2006; Meneghetti et al., 2021), but they have mainly focused on cognitive factors associated with navigation ability (Hegarty et al., 2006; Meneghetti et al., 2022). Less systematically examined are the behaviors or the uses of external aids during navigation. Indeed, in everyday life, it is possible to use social (people) and technological aids, thus changing individuals’ involvement in decision-making during navigation (Dalton et al., 2019).

The GPS tools and applications provide detailed instructions, such as spoken cues and visual arrows or maps on a display, as people or vehicles move. Due to their characteristics, devices with GPS constitute external representation that aids navigation by reducing the cognitive effort involved (Dalton et al., 2019; Wiener et al., 2009).

Given that people’s reliance on GPS technology continues to grow as GPS devices’ accuracy and usability increase, it has become relevant to better understand the relationships among GPS tools, our navigation ability, and our knowledge of the environment. Although GPS devices aid navigation and help users reach destinations (e.g., Vaez et al., 2020), they appear to have long-term detrimental effects on individuals’ navigation abilities (e.g., Dahmani & Bohbot, 2020; Javadi et al., 2017; McKinlay, 2016). Studies have shown a negative relationship between GPS use while learning an environment and subsequent recall of environmental knowledge (Dahmani & Bohbot, 2020; Ishikawa, 2019; Ishikawa et al., 2008; Münzer et al., 2012) On the other hand, it should be noted that in some cases participants using GPS were able to remember landmarks better than the group that did not use GPS tools, suggesting that spatial acquisition is still possible during GPS use for orientation (Sönmez & Önder, 2019).

In another piece of literature on navigation assistance, the researchers investigated GPS use for orientation in relation to the assessment of one’s abilities through self-reported measures (e.g., sense of direction). Most of the studies adopted self-reported measures of GPS use and sense of direction, finding that people with higher GPS use or dependency on GPS showed a weaker sense of direction (e.g., Dahmani & Bohbot, 2020; He & Hegarty, 2020; Ishikawa, 2019; Miola et al., 2023). Although it is not possible to infer a direction of causality, these findings may suggest that people with a low sense of direction may prefer to use GPS more, probably as a form of reassurance for spatial situations and navigation in daily life. The negative effect of GPS use on knowledge of the environment and sense of direction was also confirmed by a recent systematic review on GPS use and navigation ability (Miola et al., 2024).

*GPS use and spatial inclinations* Although research on assisted navigation has grown in recent years, GPS use has been less explored in relation to "spatial inclinations"—beliefs about one's spatial abilities (Meneghetti et al., 2014, 2019, 2021). Spatial inclinations include a variety of attitudes and preferences regarding spatial domain, generally related to each other (De Beni et al., 2014) They typically include either evaluation of one's own abilities (sense of direction; Hegarty et al., 2002) and the pleasure of exploring unfamiliar places and taking new roads in familiar ones or more emotional-motivational aspects such as self-efficacy, i.e., individuals’ confidence to perform spatial tasks (Miola et al., 2021) as opposed to the pleasure of navigating familiar (Meneghetti et al., 2014) places and spatial anxiety such as the degree of anxiety experienced when performing spatial and environmental tasks (Lawton, 1994). There is evidence linking spatial inclinations to environmental learning (Meneghetti et al., 2019; Miola et al., 2021), but few studies have examined their relationship with GPS use. Evidence suggests that higher spatial anxiety leads to greater reliance on GPS (He & Hegarty, 2020), while spatial self-efficacy is negatively related to GPS use (Miola et al., 2023). Additionally, frequent use of GPS for turn-by-turn directions is associated with poorer sense of direction and increased spatial anxiety (Topete et al., 2024). This highlights the need for further exploration of the relationship between spatial inclinations and GPS use.

To sum up, the literature to date has focused on the use of GPS for reaching destinations (e.g., Dahmani & Bohbot, 2020; Ishikawa, 2019; Ishikawa et al., 2008); however, other types of GPS use can be undertaken before, while, and after navigating a route. GPS tools can be used not only to find a destination but also for planning a route regarding stops or timing, looking for nearby rest areas, gas stations, or bus stops (Topete et al., 2024), reviewing the journey made, and exploring the environment. Moreover, a GPS device can be used as a digital map to understand where one is in relation to a place or spatial relationships between two places and for exploring new places of interest. These common uses reflect less of a passive following of the tool to reach destinations (turn-by-turn navigation) in favor of more strategic and active uses that are less related to knowledge of the environment and reaching destinations. To the best of our knowledge, the other uses of GPS have not been thoroughly investigated, and a measurement tool for various types of GPS use is lacking.

Therefore, it would be valuable to develop a measurement to assess a variety of GPS uses and comprehend how such behaviors are related to preferences, dependency on devices and navigation learning abilities, thereby enhancing our understanding of how to effectively utilize such devices. For example, studies are lacking concerning whether strategic use of GPS is linked to our ability to learn about an environment and to self-reported inclinations, such as spatial self-efficacy and pleasure in exploring or dependence on GPS use. To fill this knowledge gap, the current paper is intended to propose a new measure of GPS uses and its psychometric proprieties (Study 1) and explore its relations with individual differences (Study 2).

Therefore, Study 1 was based on developing a questionnaire on the various and strategic uses of GPS devices, investigating their internal structure and psychometric properties: the GPS Uses Scale on the types of GPS use. Such types of use involve conditions during a trip related to planning, use as a map, and use for exploration (strategic GPS). The other type is based on the need for reaching a destination (GPS use for wayfinding; WF).

In Study 2, we investigated whether the strategic and WF-based uses of GPS devices are associated with individual factors that the recent literature has highlighted as relevant in predicting and explaining differences in navigation performance. Among these factors, we considered gender (Nazareth et al., 2019), age (Klencklen et al., 2012), emotional-motivational aspects that captures the positive and functional dimensions of spatial inclinations and preference (spatial self-efficacy and pleasure in exploring; He & Hegarty, 2020; Miola et al., 2021; Muffato et al., 2023), dependence on assisted navigation systems (Dahmani & Bohbot, 2020), and the ability to learn and remember an environment (spatial knowledge; Ishikawa, 2019).

## Study 1

In Study 1, we aimed to implement a new questionnaire that delves into various ways GPS can be used, by exploring various strategic applications of GPS devices in addition to reaching destinations. For the creation of the instrument, our starting point was a list of GPS uses from Ishikawa, (2019), who proposed major purposes of using pedestrian navigation systems, in-car navigation systems, and maps. We classified the situations Ishikawa, (2019) proposed, added new questions, and suggested a 5-point Likert scale. We expected that the new questionnaire would comprise different dimensions assessing GPS use for WF and strategic use. The scale covers four strategic uses of GPS: (1) use during a trip, (2) use for planning, (3) use as a map, (4) use for exploration, and a (5) GPS for WF use scale. The latter includes objectives such as reaching destinations, navigating unfamiliar places, and identifying routes to follow.

**Fig. 1:**
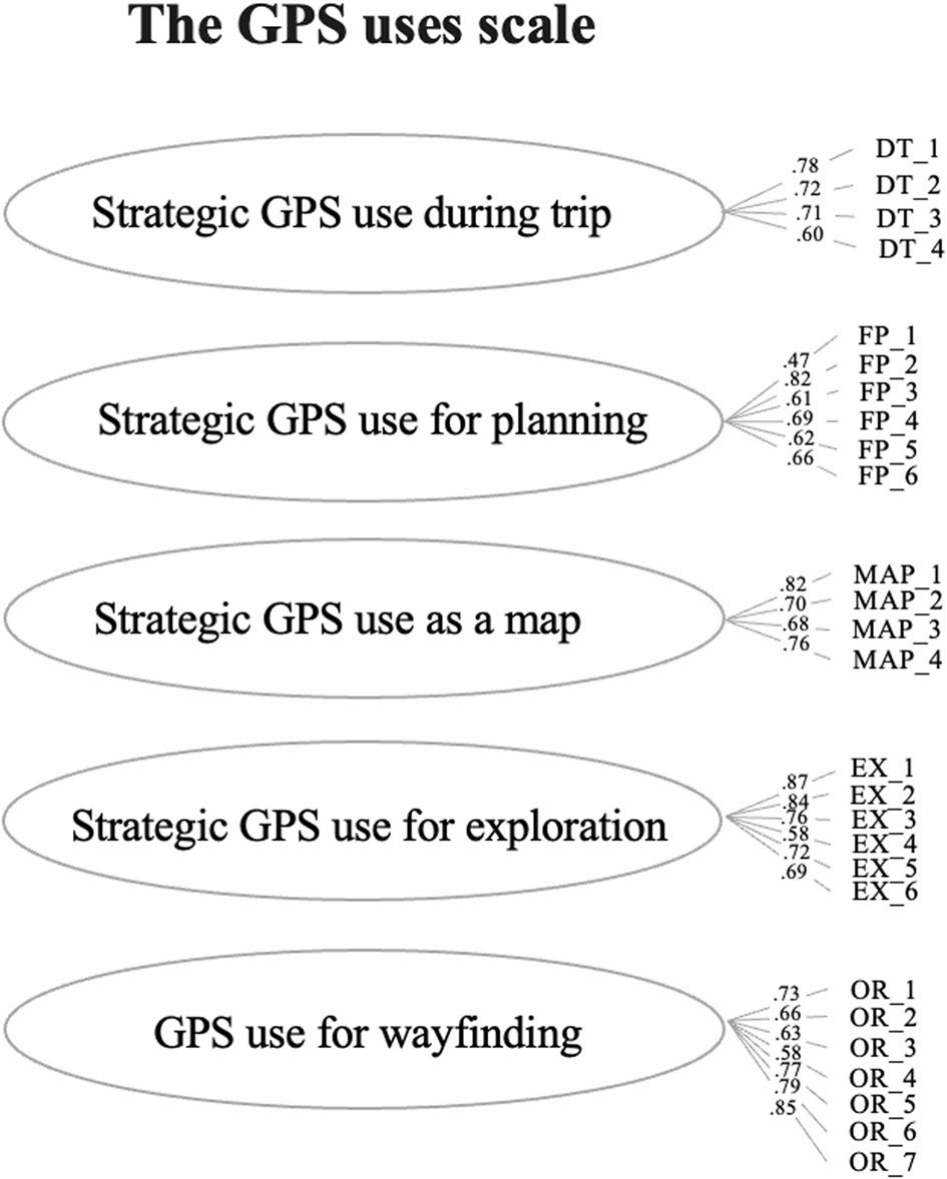
Graphical representation of the five-factor model with standardized factors loadings. *Note*: Covariances between factors are present in the model but for clarity not presented in the Figure. Covariances are from 0.30 to 0.48

The questionnaire contains in total five subscales. We tested the hypothesized structure (see Fig. 1) with confirmatory factor analysis (CFA).

A second aim of the study was to assess the new questionnaire’s reliability and validity. We used the McGill GPS questionnaire (Dahmani & Bohbot, 2020) as the most similar questionnaire available in the literature for convergent validity. The McGill questionnaire contains a reliance scale assessing the frequency of GPS use when traveling to new or previously visited routes in familiar and unfamiliar environments. Moreover, it contains a dependence scale assessing how comfortable or anxious people feel driving without GPS. In particular, we expected the GPS reliance scale of the McGill GPS questionnaire to correlate positively with the scale evaluating GPS use for orientation because both measure frequency of using GPS for orientation and wayfinding. However, we also expected correlations with the strategic subscales because those who rely more on GPS for reaching destinations may also use it more frequently for strategic behaviors. Finally, we expected the GPS dependence scale that measures GPS dependency and anxiety about traveling without GPS to correlate poorly (compared to the reliance scale) with the new questionnaire’s strategic subscales.

### Participants

A total of 365 Italian participants (229 women) between 18 and 60 years old were involved in the study (mean age = 29.74; SD = 11.29; see Table 1 for means and standard deviations divided by gender). Eleven people were eliminated because they had not fully completed the questionnaire. Of the 365 participants, 102 (51 women) between 18 and 54 years old answered the questionnaire twice for test–retest variability (mean age = 37.13; SD = 8.04). The study was approved by the local ethics committee at University of Padova (protocol No. 5327) and complied with the Declaration of Helsinki (2013). All participants gave their informed consent.

**Table 1:** Fit indices of the 3 considered factor models

	χ^2^ (df)	RMSEA	SRMR	NNFI	CFI
Model 1 (5 separate factors)	1695.33	0.09	0.09	0.96	0.97
Model 2 (2-factors; with subscales^1^)	1741 (459)	0.09	0.09	0.96	0.97
Model 3 (1-factor)	2049 (324)	0.12	0.11	0.94	0.95
Model 4 (2-factors without subscales^2^)	1824 (323)	0.12	0.10	0.95	0.95

^1^The sum of the four strategic subscale; ^2^ The sum of all items composing the four strategic subscales

### Method and materials

*Demographic questionnaire.* The demographic questionnaire encompassed items pertaining to age, gender, nationality, and occupation.

*The GPS Uses Scale* (ad hoc*).* The questionnaire concerns reasons for using GPS navigation tools. Specifically, we included the 14 items from Ishikawa, (2019) and added 18 new items. The new questionnaire contains 32 items on general GPS uses on a 5-point Likert scale (from 1 = never to 5 = always), divided into five subscales corresponding to types of use:

- *Strategic use during trip* (an example item is “How often do you use your navigator and GPS apps to search for rest areas or parking lots or gas stations or bus stops?” – 5 items).
- *Strategic use of trip planning* (an example item is “How often do you use the navigator to acquire information regarding traffic or road congestion?” – 7 items)
- *Strategic use of GPS as a map* (an example item is “How often do you use your navigator and GPS apps to understand spatial relationships between places [e.g., your current location and destination] on a map?” – 4 items)
- *Strategic use of GPS for exploration* (an example item is “How often do you use your navigator and GPS apps to discover new places to visit next?” – 6 items)
- *GPS Use for WF* (an example item is “How often do you use your navigator and GPS apps when you are lost or disoriented?” – 9 items)

We list the scale’s items in the Appendix (Italian and English version).

*McGill GPS questionnaire: GPS reliance and GPS dependence scale* (Dahmani & Bohbot, 2020). The questionnaire investigates participants’ use of GPS and lifetime GPS experience. It contains 19 items on a 5-point Likert scale related to the GPS Reliance Scale and the Sense of GPS Dependence Scale. Participants were asked to consider the past month as they answered the questions. The GPS Reliance scale contains 7 items on a 5-point Likert scale (from 1 = never to 5 = always) assessing how often people rely on GPS in various situations (e.g., “How often do you use a GPS to travel new routes to an unfamiliar destination?”, “How often do you use a GPS to travel new routes to a previously visited destination?”). The Sense of GPS Dependence Scale contains 13 items on a 5-point Likert scale (1 = strongly disagree to 5 = strongly agree) assessing the extent to which people feel dependent on their GPS (e.g., “I get lost easily in a new environment when I am not using a GPS,” “I feel anxious when driving without a GPS”). The sum of all the responses determined the final score.

#### Procedure

Participants were recruited by word of mouth and social networks. The study was conducted entirely online via Qualtrics, and questionnaires were offered in randomized order. In total, filling out the questionnaires required around 30 min. This study was part of a larger project in which participants also filled out other questionnaires. 

#### Statistical analysis

All analyses were conducted using RStudio. First, we assembled our dataset’s descriptive statistics, and we run Pearson’s correlations analysis between the subscales scores of the new GPS use questionnaire and the two scales of the McGill GPS questionnaire (reliance and dependence scale). Second, we conducted a CFA using the ‘lavaan’ package in R (Rosseel, 2012) to assess the theoretically assumed structure of the GPS Uses Scale of different subscales (Model 1; 5 different subscales of GPS use; see Fig. 1). We compared this hypothesized model with three alternative models to test various factorial structures and identify the best one. We tested a two-factor model with a strategic factor of GPS use (divided in four subscales) and a factor of GPS use for orientation and WF (Model 2; two-factor with subscales). The strategic uses of GPS include the following subscales: a) during a trip, b) for planning, c) use as a map, and d) for exploration. A single-factor model (Model 3; one-factor) that included all items together. Finally, a bifactorial model contained two factors, orientation and strategic, the latter including all strategic items together (Model 4; two-factor without subscales).

The goodness of fit was assessed with the following indices: standardized root-mean-square residual (SRMR, range 0–1), root-mean-square error of approximation (RMSEA, range 0–1), comparative fit index (CFI, range 0–1), and non-normed fit index (NNFI, range 0–1).

Convergent validity was established by correlating the GPS Uses Scale with the McGill GPS questionnaire (Dahmani & Bohbot, 2020). Moreover, test–retest reliability using data collected at baseline and at the 3-week follow-up point was calculated using Pearson’s *r* (N = 102).

### Results

#### Factor composition, reliability, and validity of the GPS uses scale

Both the five-factor model (Model 1) and the model including 2 factors with subscales (Model 2) showed better fit than the alternative models, which were therefore rejected (see Table 1). Since the subscales are all similarly correlated (see Table 2), we decided to consider the model with 5 different subscales: strategic use during a trip, for planning, use as a map, for exploration, and for orientation (Model 1). It showed the following fit indices: χ^2^(459) = 1741, RMSEA = 0.09, SRMR = 0.09, CFI = 0.97, NNFI = 0.96**.** To further improve the fit indices, we deleted five items with lower loadings (lower than 0.55; one item on the subscale of strategic use during trip, one on strategic use for planning [routes and time], and two items on GPS use for orientation). Moreover, we added the covariance of three items in the same subscale, as shown by modification indexes (Beaujean, 2014). The final version of the questionnaire contained 27 items that showed improved fit indexes of the 5 factors model: χ^2^(312) = 891.46, RMSEA = 0.07, SRMR = 0.07, CFI = 0.98, NNFI = 0.98. Figure 1 shows standardized factor loadings.

**Table 2:** Descriptive statistics of the GPS Uses Scale (27 items). Correlations with McGill GPS scale and Cronbach’s alpha

	M	SD	(1)	(2)	(3)	(4)	(5)	(6)	Cronbach alpha
1. GPS Uses Scale—Strategic GPS use during trip	11.49	3.55							0.75
2. GPS Uses Scale—Strategic GPS use for planning	23.56	5.18	0.69^***^						0.77
3. GPS Uses Scale—Strategic GPS use as a map	12.15	3.88	0.61^***^	0.70^***^					0.81
4. GPS Uses Scale—Strategic GPS use for exploration	12.63	4.76	0.50^***^	0.56^***^	0.84^***^				0.84
5. GPS Uses Scale—GPS use for orientation	24.67	5.92	0.59^***^	0.67^***^	0.61^***^	0.41^***^			0.85
6. McGill GPS reliance	22.79	4.43	0.47^***^	0.46^***^	0.362^***^	0.20^***^	0.60^***^		0.74
7. McGill GPS dependence	42.09	9.29	0.25^***^	0.21^***^	0.08	0.02	0.33^***^	0.53^***^	0.80

The McGill GPS reliance scale was included in the study to provide a convergent validity measure of the new scale. The McGill GPS reliance scale was significantly and positively correlated with strategic GPS use during a trip (*r* = 0.47, 95% CI[0.39–0.55], *p* < 0.001), for planning (*r* = 0.46, 95% CI[0.38–0.54], *p* < 0.001), as a map (*r* = 0.36, 95% CI[0.27–0.45], *p* < 0.001), for exploration (*r* = 0.20, 95% CI[0.10–0.29], *p* < 0.001), and for WF (*r* = 0.60, 95% CI[0.58–0.70], *p* < 0.001).

The McGill GPS dependence scale was significantly correlated with strategic GPS use during a trip (*r* = 0.25, 95% CI [ 0.15 –0.34], *p* < 0.001), for planning (*r* = 0.21, 95% CI [0.11–0.30], *p* < 0.001), and for WF (*r* = 0.32, [95% CI [ 0.27 –0.45], *p* < 0.001). No significant correlations emerged for use as a map (*r* = 0.08, 95% CI [-0.01–0.18], *p* > 0.05), use for exploration (*r* = 0.02, 95% CI [-0.08–0.12], *p* > 0.05).

Test–retest reliability for each subscale showed Pearson’s correlations higher than 0.60.

Table 2 shows descriptive statistics and reliability of the GPS Uses Scale and McGill GPS questionnaire. Cronbach’s alphas were calculated for all the subscales (see Table 2) and strategic GPS factors (α = 0.91).

### Discussion

In Study 1, we aimed to develop a questionnaire on uses of GPS devices, investigating its internal structure and psychometric properties. The new questionnaire was designed to assess strategic and less conventional use of GPS: a) use during a trip, b) for planning, c) use as a map, and d) for exploration. The questionnaire also covers GPS use for WF. After testing the single-factor model and two bifactor models, the structure with 5 different subscales provided the best fit with the data, indicating that it effectively measures different types of GPS usage (see Fig. 1). Cronbach’s alpha demonstrated excellent internal consistency across the five subscales. Furthermore, regarding convergent validity, our findings showed medium to high correlation coefficients (from *r* = 0.20 to *r* = 0.60) between the GPS Uses Scale factors and the McGill GPS reliance scale. Specifically, regarding the strategic subscale, the correlations with the McGill scale were positive and small to moderate, with coefficients ranging from 0.20 to 0.47, suggesting that the more frequently people use GPS devices, the more likely they are to use them for strategic purposes. The positive, small-moderate correlation with a similar measure suggests convergent validity. In contrast, the correlations with the McGill Dependence scale were lower than the reliance scale (ranging from 0.02 to 0.25), indicating that strategic uses are only weakly associated with dependence in orientation and navigation contexts and situations.

## Study 2

Study 2 was planned to investigate whether GPS strategic uses and use for orientation—assessed with the new scale derived from Study 1—are related to individual factors. As a result of the previous study, having the opportunity to assess uses of GPS for the first time, we explored whether and how strategic and less conventional GPS Uses Scale (strategic use during trip, for trip planning, use as a map and for exploration) and use for WF (using the new scales of the GPS uses questionnaires) are correlated with individual factors, such as age and gender (given their relevant role in spatial navigation; Nazareth et al., 2019), as well as emotional-motivational aspects, such as self-efficacy and pleasure in exploring (factors shown to predict spatial navigation and knowledge; Miola et al., 2021) and the ability to learn and retain information about one’s surroundings (given that GPS use is related/supposed to be related to spatial knowledge; Dahmani & Bohbot, 2020; Ishikawa, 2019). In addition, we included the GPS dependence (Dahmani & Bohbot, 2020) as a measure of navigational behaviors and how much people trust the device. The purpose of including GPS dependency is to explore its relationship not only with motivational factors, such as self-efficacy and pleasure in exploration, but also with aspects of navigational behavior and usage patterns (He & Hegarty, 2020). To avoid adding too many similar, covarying variables as predictors, we selected only the dependence scale from the McGill GPS scale. This scale includes items related to anxiety and enables us to measure a dimension that differ from self-efficacy. Previous evidence showed a negative relation between spatial self-efficacy and GPS use for orientation (Miola et al., 2023), whereas here, we expected positive associations among strategic GPS use, spatial self-efficacy, and pleasure of exploration, highlighting the functional and positive aspects of strategic GPS use. Moreover, in line with previous studies, we expected a positive association between GPS use for orientation and GPS dependency (Dahmani & Bohbot, 2020). Concerning gender, we might hypothesize that men use GPS more strategically and less for orientation than women (Miola et al., 2023) in light of studies showing better navigation abilities attributed to men (e.g., Nazareth et al., 2019). Finally, to further explore GPS uses in daily life, we included a repeated measure of GPS uses for 10 consecutive days to have an overview of the strategic (or otherwise) uses of GPS devices, examining the frequency of type of use in everyday situations. This measurement provides a more comprehensive evaluation of daily GPS usage over an extended period, particularly for describing people's behaviors.

### Participants

Two hundred participants (98 women) between 18 and 48 years old were involved in the study (mean age = 23.52; SD = 5.48; see Table 3 for means and standard deviations). We excluded three people who did not identify as male or female because we were interested in including the gender variable. The study was approved by the local ethics committee at Authors’ University (protocol No. 172-b) and complied with the Declaration of Helsinki (2013). A power analysis was run using the pwr library for R studio, and 194 was the minimum sample size necessary to detect a small effect size (*r* = 0.20), at a significance criterion of α = 0.05 with 80% power.

**Table 3:** Correlation analysis—Study 2

	M	SD	(1)	(2)	(3)	(4)	(5)	(6)	(7)
1. GPS during trip	12.4	3.53							
2. GPS for planning	20.6	5.03	0.61^***^						
3. GPS as a map	13.3	3.88	0.46^***^	0.57^***^					
4. GPS for exploration	13.4	4.65	0.48^***^	0.50^***^	0.51^***^				
5. GPS for orientation	26.5	5.81	0.58^***^	0.63^***^	0.58^***^	0.44^**^			
6. Self-efficacy and pleasure	111.1	27.7	0.04	0.04	0.13	0.19^*^	− 0.21^***^		
7. GPS dependency	42.2	8.36	0.15^*^	0.07	0.05	− 0.08	0.37^***^	− 0.67^***^	
8. Map task	4.78	1.68	0.05	0.10	0.07	0.08	0.06	0.24^**^	− 0.16^*^

### Method and materials

#### Demographic questionnaire

The demographic questionnaire encompassed items regarding age, gender, nationality, occupation, and level of physical activity.

#### The GPS Uses Scale

See Study 1 for details (See Appendix for the questionnaire). We report the scale’s items in the Appendix (Italian and English version). For the purposes of the present study, we summed the items referring to the strategic use’s factors together, and the items for orientation and wayfinding factors. Cronbach’s alpha for the present sample was 0.90.

*McGill GPS dependence scale* (Dahmani & Bohbot, 2020). The McGill GPS Dependence scale contains 13 items on a 5-point Likert scale (1 = strongly disagree to 5 = strongly agree) assessing the extent to which people feel dependent on their GPS (e.g., “I get lost easily in a new environment when I am not using a GPS,” “I feel anxious when driving without a GPS”). The sum of all the responses determined the final score. Cronbach’s alpha for the present sample was 0.74.

#### Self-efficacy and pleasure in exploring questionnaire (Author et al., in preparation)

The questionnaire measures how confidently individuals feel about their ability to perform environmental tasks and their pleasure in exploration situations. The questionnaire contains 22 items scored on a 6-point scale (from 1 = *completely disagree* to 7 = *completely in agreement*). Example items are “I enjoy finding new ways even to reach familiar places” and “I feel able to reach an appointment location in an unfamiliar part of town”. The total score was determined by adding together all the ratings. Cronbach’s alpha for the present sample was 0.91.

#### GPS diary for 10 days (repeated measurement of GPS use)

The participants were asked to answer the same questions at the end of the day for 10 days. Each day, they recorded the date, whether they had moved from home to a familiar or unfamiliar place, and whether they had used GPS. If they used it, they indicated whether they had time pressure (“Did you use GPS because you had an appointment and wanted to be sure to arrive on time? In other words, did you use the GPS to know how to get to your destination without getting lost and arriving late?”) and the context in which they used the GPS tool (daily use [work, study, etc.], for work/study on an extraordinary occasion [e.g., a company outing for work], for pleasure [vacation, outing, etc.] outing with friends [bar, pizza place, restaurant]) and for how long. Then, the participants responded to the GPS Uses Scale, indicating among the scope they used GPS each day (among the 27 uses of the questionnaire).

#### Virtual environment learning and environment recall tasks

**Fig. 2:**
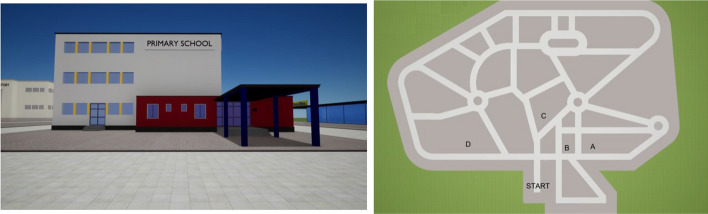
Example of the map task: landmarks (on the left) and sketch map (on the right) shown to participants

*Encoding phase* An outdoor area consisting of a virtual urban city (Miola et al., 2021) with 19 landmarks (e.g., school, bank, park, fountain, statue) and a grid of streets with three roundabouts comprised the study’s virtual environment (for virtual environment details, see the authors’ linked paper, Miola et al., 2021 and Fig. 2). The image of the video was created at the eye height of 160 cm, and the camera was set with a horizontal field of view of 90◦. Rotation and translation settings were fixed for all participants. The walking speed was 4 m/s.

During the learning phase, participants watched a first-person video of a person moving through a route of the virtual city that was roughly one kilometer long. The video lasted around 4 min and is projected on the desktop screen. After watching it twice, participants were instructed to focus on every aspect of the surroundings, including the buildings, route, and layout.

*Environment recall task: the sketch map task* After the learning phase, participants performed an online task, the sketch map task, which measures the ability to identify relationships between the location of various elements in the environment irrespective of the observer’s previous view of the environment. Specifically, participants were shown an image representing a landmark together with a map of the environment with the starting point and 4 possible locations marked with the letters A, B, C, and D (see Fig. 2). Participants indicated where the landmark was located. The task comprised 7 items. One point was awarded for each right answer and zero for wrong answers; higher scores indicated greater ability in the map task. The maximum score was 7. Cronbach’s alpha for the present sample was 0.57.

#### Procedure

The procedure was divided into two online sessions in Qualtrics. The first session consisted of self-completion of questions and questionnaires on a) demographic characteristics (age, gender, age profession, sport), b) self-efficacy and pleasure of exploring environments, c) the GPS Uses Scale, and d) McGill GPS dependence scale, in that order. Afterward, e) the participant watched an online video of a route in a virtual environment twice. At the end of the second viewing of the video (about 8 min), participants performed an online map task, such as locating on a sketch map the correct position of 7 landmarks. The completion of the questionnaires and map task in Session 1 was balanced among the participants: half of the participants started with the questionnaires, and half started with the navigation test.

The following day, in session 2, participants were instructed to fill out the questionnaire at the end of the day about the type of use for each time they used GPS over 10 days. Every day, the investigator sent participants a reminder to complete the questionnaire via email. The ten-day assessment started on Wednesday or Thursday and included one weekend.

#### Statistical analysis

All analyses were conducted using RStudio. First, we assembled the descriptive statistics of our dataset, including the questionnaires and the repeated measurement of GPS use (GPS diary use). Then, linear models were computed to estimate individual factors’ effects on the following dependent variables: a) strategic uses of GPS (considered here as together for clarity of results presentation) and b) GPS use for WF scale as assessed by the GPS Uses Scale (Study 1). The model included gender, age, self-efficacy, GPS dependency, and map task (m0). Using AIC-based stepwise model selection, we removed the predictors one by one to select the best-fitting model and to diminish overfitting. For both the dependent variables, four models were compared. Before the models were computed, all variables were standardized. Through the repeated measurement of GPS use, the rates of GPS use during the 10-day period were calculated (see Tables 5–9).

### Results

Table 3 provides descriptive statistic, and Table 4 details linear models.

**Table 4:** Models considered in the model comparison procedure for each dependent variable

	Model	AIC	R^2^
*Strategic use of GPS*			
**M0**	**Strategic Uses ~ Gender + Age + Self-efficacy + GPS dependency + Map task**	**564**	**0.05**
M1	Strategic Uses ~ Gender + Age + Self-efficacy + GPS dependency	564	0.05
M2	Strategic Uses ~ Gender + Age + Self-efficacy	569	0.02
M3	Strategic Uses ~ Gender + Age	570	0.01
*GPS use for orientation*			
**M0**	**Use for WF ~ Gender + Age + Self-efficacy + GPS dependency + Map task**	**540**	**0.16**
M1	Use for orientation ~ Gender + Age + Self-efficacy + GPS dependency	542	0.15
M2	Use for orientation ~ Gender + Age + Self-efficacy	561	0.06
M3	Strategic Use ~ Gender + Age	568	0.02

Best models (lower AIC) are highlighted in bold

*Strategic GPS use* The stepwise AIC procedure suggested that the model that most improved the AIC index (best AIC = 564) included gender, age, self-efficacy, dependence, and map task performance (M0 and M1 showed the same AIC value; we chose M0 as it consists of our hypothesis driven model). All other AICs were greater than 564. The hypothesized predictors accounted for the 5% of the variance calculated using the adjusted R^2^ [*F* (5, 195) = 3.15, *p* = 0.009]. A statistically significant main effect of self-efficacy (*B* = 0.27, CI [0.08, 0.47],* p* = 0.004) and GPS dependence (*B* = 0.24, CI [0.06, 0.43],* p* = 0.009) suggested that self-efficacy and dependence on GPS devices positively predicted the strategic use of GPS. Outliers were diagnosed using Cook’s distance value. Cook’s distance values were all lower than 1 (mean Cook’s distance = 0.005599). Multicollinearity was examined using the variance inflation factor (VIF), which ranged from 1.02 to 1.88, indicating low-moderate collinearity.

Cronbach’s alpha in the present sample for strategic GPS use is 0.86.

*GPS for WF* The model that most improved the AIC index (best AIC = 540) included gender, age, self-efficacy, dependence, and map task performance (M0). All other AICs were greater than 540. The predictors accounted for 16% of the variance calculated using the adjusted R^2^ [*F* (5, 194) = 8.68, *p* < 0.001]. A statistically significant main effect of gender (*B* = -0.31, CI [-0.57, -0.05],* p* = 0.01), dependence on GPS (*B* = 0.41, CI [0.24, 0.58],* p* < 0.001), and map task (*B* = 0.14, CI [0.002, 0.26],* p* = 0.045) suggests that men use GPS devices less frequently than women for orientation purposes. Moreover, the dependence on GPS and the ability to mentally represent the environment positively predicted the use of GPS for orientation. Outliers were diagnosed using Cook’s distance value. Cook’s distances values were all lower than 1 (mean Cook’s distance = 0.004604). Multicollinearity was examined using the VIF, which ranged from 1.02 to 1.96, indicating low-moderate collinearity.

Cronbach’s alpha in the present sample for strategic GPS use is 0.78.

*GPS diary* Finally, we calculated the proportion of GPS use in familiar and unfamiliar environments by considering the number of times one reports having moved from home (see Table 5). Then we calculated the proportions of GPS use in diverse contexts and situations (e.g., daily use or occasions; Table 6) and the proportion of use for each questionnaire item (27 items; Table 7). Finally, we present two tables showing the uses the participants indicated most frequently for familiar and unfamiliar environments (above 5%; Tables 8 and 9). We correlated GPS use frequency and type of use in 10 days with the score on the GPS Uses Scale discussed in session 1 and was not significant.

**Table 5:** Percentage of GPS use overall and percentage of use in familiar and non-familiar environment based on the reported numbers of travels from home in the 10 days

	Percentage (%)
GPS use in 10 days	24.48
GPS use in familiar environment	35.3
GPS use in unfamiliar environment	64.5

**Table 6:** Percentage of GPS use in different context situations in 10 days

Context of using GPS devices	Percentage (%)
1. Work/study on an extraordinary occasion [e.g., a company outing for work]	18.4
2. Pleasure [vacation, outing, etc.]	21.5
3. Outing with friends [bar, pizza place, restaurant])	27.6
4. Daily use (work, study, etc.)	26.9
NA	5.6

**Table 7:** Percentage of GPS uses in 10 days (Strategic use of GPS devices questionnaire; 27 items) from highest to lowest percentage of the total number of times people traveled from home

Use of GPS (items of the scale)	Percentage (%)
To look up the address of a place	14.6
When traveling to an unfamiliar place (visiting for the first time)	12
To know how long it takes to reach a place	8.18
To know where a destination is located	7.67
To know, be shown, the route to a destination	6.91
To figure out in which direction is the place to be reached	6.65
To choose the fastest route based on traffic	5.37
To look at the route before leaving	3.58
To check that the place (e.g., store) I am going is open	3.32
When one is lost or disoriented	2.81
For long stretches, on extended trips	2.56
To anticipate turns and curves on the road I am traveling on	2.30
To understand the spatial relationships between places (e.g., its current location and destination) on a map	2.05
To discover new places to visit later	1.79
To estimate the distance from one place to another	1.53
To know where you are	1.53
To plan routes with multiple stops or destinations	1.28
To look for an alternative route, when, for example, encountering road congestion or construction work	1.28
To search for rest areas, parking lots, gas stations or bus stops	1.02
Based on distance to determine which means of transportation to use	0.76
To see where I am from the map (geolocation)	0.76
To search for places of interest nearby	0.76
To explore a preview of the place where I am	0.51
To search for the name of a place	0.25
To learn the names of streets	0.25
To virtually retrace streets to find places discovered by chance	0.25
To remember where I have been	0.25

**Table 8:** Proportion (more than 5%) of GPS use in 10 days in FAMILIAR environment, from largest to smallest of the total number of times people traveled from home

Uses of GPS – FAMILIAR environment	Percentage (%)
To know how long it takes to reach a place	13
To check that the place (e.g., store) where I am going is open	7.9
To know where a destination is located	7.9
To know, be shown, the route to a destination	5.8

**Table 9:** Proportion (more than 5%) of GPS use in 10 days in UNFAMILIAR environment, from largest to smallest of the total number of times people traveled from home

Uses of GPS – UNFAMILIAR environment	Percentage (%)
To look up the address of a place	20.6
When traveling to an unfamiliar place (visiting for the first time)	16.3
To figure out in which direction is the place to be reached	7.94
To know, be shown, the route to a destination	7.54
To know where a destination is located	7.54
To choose the fastest route based on traffic	6.75
To know how long it takes to reach a place	5.56

### Discussion

Study 2 was intended to examine the relationship between different types of GPS uses—more strategic use and use for WF—and individual differences in terms of gender and age, spatial inclinations and behaviors (self-efficacy and GPS dependency), and the ability to learn and retain information about one’s surroundings (environmental knowledge). The types of use were also monitored for 10 consecutive days.

The relationships between GPS use and individual factors were tested with linear models to determine whether various predictors can have a role in the two aspects of GPS use (strategic and for WF). Interestingly, the best-fitting model includes all predictors for both strategic GPS use and GPS use for orientation suggesting that each of the individual factors considered contributes to explain both types of GPS usage and that they are all statistically relevant. Overall, the hypothesized predictors account for 5% of the variance in strategic GPS use, while explaining 16% of the variance in GPS use for orientation. These findings underscore that the predictors hypothesized explain especially GPS usage for orientation and WF purposes. Concerning the statistical significance for strategic use of GPS, results showed that self-efficacy and pleasure in exploring and GPS dependence emerged as significant predictors: people with high self-efficacy and pleasure in exploring tend to use the GPS in more strategic ways, such as for traveling, searching for places with the environment around them, and planning the route. Moreover, people with high dependency on GPS for orientation use it more also in a strategic way.

Regarding GPS use for WF, gender, dependence on GPS, and map task accuracy emerged as significant predictors: men tend to use GPS devices more often, and people who reported higher dependence on GPS and greater ability to mentally represent an environment showed more frequent GPS use for orientation.

Overall, these results reveal that feeling more effective and confident in spatial situations, predicts the GPS use for strategic purposes but not for WF, highlighting the positive role of self-efficacy for strategic use and the difference between the strategic and for wayfinding uses. Moreover, it appears that feeling dependent on the device is linked to both types of usage, suggesting that device dependency could lead to its use for WF purposes but also for more strategic navigation.

The results on gender confirm previous findings in the literature that men tend to use GPS for orientation less than women (e.g., Miola et al., 2023). Finally, mental representation ability (as measured by a sketch map task) is positively associated with the use of GPS for WF. This result was unexpected as other studies have not found a relationship between GPS reliance and performance in virtual navigation (Yavuz et al., 2024) or using GPS while learning an environment seems to affect the configural representation of it (e.g., Ishikawa, 2019). However, a possible explanation could lie in the fact that the scale of GPS use for WF contains items especially with respect to unfamiliar locations so even those with good skills might use it while traveling and navigating in unknown places. As regards of the use of GPS over 10 days, the GPS device is utilized in a similar way for social events, going out with friends, and for more regular purposes, such as getting to work. Furthermore, two strategic uses and two orientation uses are the four most common uses of GPS in familiar situations. Conversely, in unfamiliar locations, three of the seven most common uses of GPS were strategic. These findings demonstrate that over a period of ten days, the proportions of more strategic and oriented activities were similar; however, in familiar environments uses are more on time planning while in unfamiliar are more orientation and space purposes. Overall, the most frequently mentioned uses were “to look up the address of a place” and “to know how long it takes to reach a place.”

## General discussion

Navigation is a complex ability related to several factors (Hegarty et al., 2006; Wiener et al., 2009). The use of technologies, devices, and apps to support orientation and navigation abilities is increasing. Devices and apps take advantage of GPS, allowing people to access a variety of spatial information at any time to reach a destination. It should be noted that the questionnaires for detecting the use of GPS available in the literature focus on uses to orient oneself, finding destinations or on how dependent and comfortable the person feels in navigating with devices or not (e.g., Dahmani & Bohbot, 2020). Today, with the increasing technology use for everyday moves, assessing the dependency is limitative.

Indeed, there are various GPS uses that are independent of the knowledge of the environment orientation or wayfinding purposes. One of the first works that proposed a variety of GPS uses is Ishikawa’s (2019), which relied on a descriptive questionnaire. Inspired by this work, we outlined uses that can be considered for strategic purposes in the context of navigation (and orientation) or independent from reaching a destination. Examples of strategic purposes are planning a route and estimating travel time, locating parking lots or bus stops along the way, exploring the surrounding environment, and understanding spatial relationships between landmarks. Dimensions of navigation include various steps, from planning to execution or to revision of the route (Wiener et al., 2009), and strategic use of GPS can be involved in these phases. Other uses involve time planning in relation to route planning. Additionally, there is a lack of a comprehensive tool, such as a questionnaire, that encompasses various uses of GPS beyond mere navigation to destinations. Such a tool is essential for studying individual differences in GPS behaviors and to understand how to best enhance the usage of these devices for people. Therefore, we implemented a questionnaire intended to measure the various types of GPS use, considering use for orientation and, on the other hand, for planning, exploring, and understanding spatial relationships (strategic use). We thus created a valid and reliable measure of GPS use and investigated its internal structure and psychometric properties (Study 1). Subsequently, with a second study (Study 2), we explored the relationship between GPS use and individual factors such as age, gender, and self-reported aspects of navigation (self-efficacy and pleasure of exploring and GPS dependency) and environment knowledge (sketch map task).

As in Study 1, we outlined 5 subscales of the questionnaires: (1) strategic use during trip, (2) strategic use of trip planning, (3) strategic use of GPS as a map, and (4) strategic use of GPS for exploration (5) GPS use for orientation and WF**.** Starting with 32 items (including Ishikawa’s items), the final questionnaire comprised 27 items distinguished in two factors showing acceptable fit indexes. The questionnaire showed a convergent validity (McGill GPS questionnaire-Reliance Scale) and validity stable across time (3 weeks).

The study’s novelty lies in the introduction of the concept of various type of uses including strategic GPS utilizations, which goes beyond reliance solely on reaching destinations or orientation purposes. However, it is important to point out that the types of uses do correlate positively each other. Although the best factorial structure in our data shows that these uses are different, suggesting five different nuances (four strategic and one of orientation), they are still related. Thus, the greater the use for orientation and WF, the greater the strategic uses and vice versa.

In Study 2, the new questionnaire was used to explore how the types of GPS usage relate to individual differences such as gender and age, spatial inclination and behaviors, and the ability to learn and retain information about one’s surroundings environment knowledge (cognitive ability). The results revealed that different factors come into play for strategic uses and for orientation use.

As for strategic uses of GPS, those who reported greater self-efficacy and pleasure in exploring the environment tend to use GPS for strategic purposes (positive relationship). This interesting result highlights that these types of GPS use are related to people’s personal inclination to explore the environment in certain spatial situations and feel effective, highlighting the positive nature of these types of device use. Following this result, it is interesting to point out that self-efficacy and pleasure in exploring, on the other hand, correlates negatively with the use of GPS for WF (from correlational analyses) accordingly to previous evidence showing that frequency of GPS use in general is negatively correlated with self-efficacy and positively with spatial anxiety (He & Hegarty, 2020; Miola et al., 2023).

Moreover, results revealed that those who are more dependent on GPS devices in general tend to use it for different purposes, including more strategic ones. This result suggests that the extent to which people feel dependent and anxious on their GPS can be related to the amount of GPS use, even when GPS use is strategic and less related to navigation. The more people feel dependent on GPS devices, the more they use it also for strategic purposes.

No relationship emerged for other predictors, such as gender, age, and environment knowledge. Overall, the results newly suggest that it is not so much spatial cognitive skills (environmental knowledge) that affects the strategic uses of GPS but rather the spatial inclinations and behaviors.

Regarding GPS use for orientation, the results from linear models showed that gender, dependence on GPS, and environmental knowledge (sketch map accuracy) emerged as significant predictors. The findings suggest that being a man was negatively related to the use of GPS devices for orientation. This result aligns with those of Miola et al., (2023), which compared men’s and women’s frequency of GPS use and found that men use GPS devices for orientation less often than women. These results also align with evidence showing that men, on average, have better large-scale navigation abilities than women (e.g., Nazareth et al., 2019), and consequently suggesting that men could use fewer external aids for navigation. However, there is also no major use of GPS in a strategic way by men, as hypothesized, which may indicate that men, on average, probably rely on their abilities when navigating.

Furthermore, those who are more dependent on GPS, and feel anxious without it, reported using GPS for orientation more often. Considering the bidirectionality of the relationship, those who use GPS devices more often for orientation may also be more anxious when they do not have the devices and feel more dependent on them. Finally, those having greater cognitive ability to create a mental representation of the environment (assessed with the sketch map task) use GPS more for orientation purposes. Such evidence may seem not aligned with previous studies showing that the frequency of GPS use in various scenarios is negatively related to the ability to learn an environment (e.g., Topete et al., 2024) or that found no significant relationships (Yavuz et al., 2024); however, it is important to note that the questions in our new questionnaire (GPS use for orientation) concerns unfamiliar environments and traffic situations (e.g., “when traveling to an unfamiliar place,” “when one is lost or disoriented,” “to look for an alternative route, when, for example, encountering road congestion or construction work”) instead of dependence on GPS in a familiar environment. Therefore, for these kinds of orientation uses of GPS, which are more functional, one’s ability to know the environment is positively associated with GPS use. This result therefore highlights the more positive characteristics that the items of this new questionnaire capture, suggesting that the greater one’s general ability to create a mental representation of an environment (environmental knowledge), the more often they will use the GPS for orientation, especially in unfamiliar environments (items referring to GPS use for orientation purposes mainly referred to unfamiliar environments).

On the other hand, considering correlation analysis, the use of GPS for orientation is negatively associated with spatial self-efficacy, suggesting that those with higher self-efficacy during exploration tend to use fewer GPS devices for navigation. These results overall showed that there is an apparent trade-off between beliefs (spatial self-efficacy and pleasure in exploring) and environment knowledge (map accuracy), which warrants further examination in future studies.

Another result emerges from the descriptive data of GPS use for 10 days. Repeated data collection for 10 days, including the weekend, gave a broader picture of people’s GPS use in the present study. The purpose of this measure was to monitor and better understand people's daily use. In general, the results show that only 1 out of 4 (25%) times people moved from home and then went out, they reported to use the GPS. The GPS device is similarly used for occasions of pleasure, such as going out with others, and for more everyday use, such as reaching one’s workplace. GPS devices are therefore not used especially for less frequent or unusual events and tasks but on a daily basis for everyday activities. Moreover, we explored for what purposes the participants used GPS devices in familiar and unfamiliar places given the different uses in these different environments. The descriptive percentage regarding navigating in familiar environment show that GPS devices are used for knowing how long it takes to reach a place (13%) and to check whether the place (e.g., store) where people are going is open (7.9%). These two purposes are among the strategic uses, therefore when knowledge of the environment has already been built up to a large extent—as in a familiar environment—people use GPS to monitor time rather than spatial information.

While, people frequently use GPS devices in unfamiliar environments (64%) to look up the address of a place (20.6%), when traveling to an unfamiliar place (visiting for the first time, 16.3%), and to figure out in which direction is the place to be reached (7.94%). Overall, besides in unfamiliar environment the percentage show some equity between strategic and orientation behavior we can state that it is especially behavior aimed at acquiring spatial information.

Despite the study’s novelty, there are some limitations to be mentioned. One limitation of the current study is the use of self-report measures and the correlational nature of the study. Based on the data presented, we are unable to draw any conclusive causal statements regarding the relationship between navigation ability, spatial inclinations, and GPS uses.

Another limitation might stem from the administration of the 10-day measurement because it would have been more reliable to administer the questionnaire with randomized items. Without randomization, there may have been a tendency for people to respond to the first items they encountered on the questionnaire.

Future studies could further explore the influence of age and gender, as well as their interaction, on GPS use. Another important area for future investigation could be an experimental study, incorporating a manipulation, to examine the effects of strategic GPS uses on navigation and environment knowledge.

ln conclusion, the study proposes a new scale to investigate the uses of GPS by paying attention to positive and strategic aspects of this navigation behavior. Navigational phases indeed expose people to strategic usage of GPS, such as planning ahead and anticipating specific activities to maximize time efficiency and ensure destination arrival. This use of GPS engages the user and enables spatial problem solving (better planning, more effective handling of unforeseen circumstances); indeed, it is associated with high self-efficacy and pleasure in exploring, highlighting that GPS use is not necessarily negative but may help in everyday life by enhancing exploration and strategic navigation behavior. We then present a new assessment of uses, outlining strategic and useful components to navigation for the first time.

## Data Availability

Data are available on request.

## Appendix

### Italian version of the GPS Uses Scale

Istruzioni: *Quanto spesso usi il navigatore e applicazioni con il GPS per i seguenti scopi:*

1 = mai, 2 = a volte, 3 = spesso, 4 = molto spesso, 5 = sempre

### Uso strategico durante il viaggio

1-Per cercare l’indirizzo di un luogo.

1 = mai, 2 = a volte, 3 = spesso, 4 = molto spesso, 5 = sempre.

2-Per cercare il nome di un luogo.

1 = mai, 2 = a volte, 3 = spesso, 4 = molto spesso, 5 = sempre.

3-Cercare aree di sosta o parcheggi o benzinai o fermate dell’autobus.

1 = mai, 2 = a volte, 3 = spesso, 4 = molto spesso, 5 = sempre.

4-Per anticipare svolte e curve della strada che sto percorrendo.

1 = mai, 2 = a volte, 3 = spesso, 4 = molto spesso, 5 = sempre.

### Pianificazione (percorso e tempo)

5-Scegliere la strada più veloce in base al traffico.

1 = mai, 2 = a volte, 3 = spesso, 4 = molto spesso, 5 = sempre.

6-Per sapere quanto tempo si impiega per raggiungere un luogo.

1 = mai, 2 = a volte, 3 = spesso, 4 = molto spesso, 5 = sempre.

7-Per programmare percorsi con più tappe o destinazioni.

1 = mai, 2 = a volte, 3 = spesso, 4 = molto spesso, 5 = sempre.

8- Per guardare il percorso prima di partire. 1 = mai, 2 = a volte, 3 = spesso, 4 = molto spesso, 5 = sempre.

9-Per controllare che il posto (ad esempio negozio) dove sto andando sia aperto.

1 = mai, 2 = a volte, 3 = spesso, 4 = molto spesso, 5 = sempre.

10-In base alla distanza valutare quale mezzo utilizzare.

1 = mai, 2 = a volte, 3 = spesso, 4 = molto spesso, 5 = sempre.

### GPS come mappa

11-Per valutare la distanza da un luogo all’altro.

1 = mai, 2 = a volte, 3 = spesso, 4 = molto spesso, 5 = sempre.

12-Capire verso quale direzione è il luogo da raggiungere.

1 = mai, 2 = a volte, 3 = spesso, 4 = molto spesso, 5 = sempre.

13-Vedere dove mi trovo dalla mappa (geolocalizzazione).

1 = mai, 2 = a volte, 3 = spesso, 4 = molto spesso, 5 = sempre.

14-Per capire le relazioni spaziali tra luoghi (e.g., la sua località attuale e la destinazione) *su una* mappa.

1 = mai, 2 = a volte, 3 = spesso, 4 = molto spesso, 5 = sempre.

### Esplorazione

15-Per cercare posti di interesse nei dintorni.

1 = mai, 2 = a volte, 3 = spesso, 4 = molto spesso, 5 = sempre.

16-Scoprire posti nuovi da visitare successivamente.

1 = mai, 2 = a volte, 3 = spesso, 4 = molto spesso, 5 = sempre.

17-Esplorare in anteprima il luogo in cui mi trovo.

1 = mai, 2 = a volte, 3 = spesso, 4 = molto spesso, 5 = sempre.

18-Imparare i nomi delle vie.

1 = mai, 2 = a volte, 3 = spesso, 4 = molto spesso, 5 = sempre.

19-Ripercorrere virtualmente delle strade per ritrovare luoghi scoperti per caso.

1 = mai, 2 = a volte, 3 = spesso, 4 = molto spesso, 5 = sempre.

20-Per ricordarmi dove sono stato.

1 = mai, 2 = a volte, 3 = spesso, 4 = molto spesso, 5 = sempre.

### Uso per orientamentoe wayfinding

21-Quando si viaggia in un luogo non familiare (che si visita per la *prima* volta).

1 = mai, 2 = a volte, 3 = spesso, 4 = molto spesso, 5 = sempre.

22-Per lunghi tratti, in viaggi prolungati.

1 = mai, 2 = a volte, 3 = spesso, 4 = molto spesso, 5 = sempre.

23-Per conoscere, farsi indicare, il percorso verso una destinazione.

1 = mai, 2 = a volte, 3 = spesso, 4 = molto spesso, 5 = sempre.

24-Per cercare un tragitto alternativo, quando, ad esempio, si incontrano congestioni stradali o lavori in corso.

1 = mai, 2 = a volte, 3 = spesso, 4 = molto spesso, 5 = sempre.

25-Quando si è persi o disorientati.

1 = mai, 2 = a volte, 3 = spesso, 4 = molto spesso, 5 = sempre.

26-Per sapere dove ci si trova.

1 = mai, 2 = a volte, 3 = spesso, 4 = molto spesso, 5 = sempre.

27-Per sapere dove si trova una destinazione.

1 = mai, 2 = a volte, 3 = spesso, 4 = molto spesso, 5 = sempre.

## English translation of The GPS Uses Scale

Instructions: *How often do you use the navigator and applications with GPS for the following purposes:*

### Strategic use during trip

1-To look up the address of a place.

1 = never, 2 = sometimes, 3 = often, 4 = very often, 5 = always.

2-To search for the name of a place.

1 = never, 2 = sometimes, 3 = often, 4 = very often, 5 = always.

3-To search for rest areas, parking lots, gas stations, or bus stops.

1 = never, 2 = sometimes, 3 = often, 4 = very often, 5 = always.

4-To anticipate turns and curves on the road I am traveling on.

1 = never, 2 = sometimes, 3 = often, 4 = very often, 5 = always.

### Strategic use for planning (routes and time)

5-To choose the fastest route based on traffic.

1 = never, 2 = sometimes, 3 = often, 4 = very often, 5 = always.

6-To know how long it takes to reach a place.

1 = never, 2 = sometimes, 3 = often, 4 = very often, 5 = always.

7-To plan routes with multiple stops or destinations

1 = never, 2 = sometimes, 3 = often, 4 = very often, 5 = always.

8-To look at the route before leaving.

1 = never, 2 = sometimes, 3 = often, 4 = very often, 5 = always.

9-To check that the place (e.g., store) I am going is open.

1 = never, 2 = sometimes, 3 = often, 4 = very often, 5 = always.

10-Based on distance to determine which means of transportation to use.

1 = never, 2 = sometimes, 3 = often, 4 = very often, 5 = always.

### GPS used as a map

11-To estimate the distance from one place to another.

1 = never, 2 = sometimes, 3 = often, 4 = very often, 5 = always.

12-To figure out in which direction is the place to be reached.

1 = never, 2 = sometimes, 3 = often, 4 = very often, 5 = always.

13-To see where I am from the map (geolocation).

1 = never, 2 = sometimes, 3 = often, 4 = very often, 5 = always.

14-To understand the spatial relationships between places (e.g., its current location and destination) on a map.

1 = never, 2 = sometimes, 3 = often, 4 = very often, 5 = always.

### Strategic use for exploration

15-To search for places of interest nearby.

1 = never, 2 = sometimes, 3 = often, 4 = very often, 5 = always.

16-To discover new places to visit later.

1 = never, 2 = sometimes, 3 = often, 4 = very often, 5 = always.

17-To explore a preview of the place where I am.

1 = never, 2 = sometimes, 3 = often, 4 = very often, 5 = always.

18-To learn the names of streets.

1 = never, 2 = sometimes, 3 = often, 4 = very often, 5 = always.

19-To virtually retrace streets to find places discovered by chance.

1 = never, 2 = sometimes, 3 = often, 4 = very often, 5 = always.

20-To remember where I have been.

1 = never, 2 = sometimes, 3 = often, 4 = very often, 5 = always.

### GPS use for orientation

21-When traveling to an unfamiliar place (visiting for the first time).

1 = never, 2 = sometimes, 3 = often, 4 = very often, 5 = always.

22-For long stretches, on extended trips.

1 = never, 2 = sometimes, 3 = often, 4 = very often, 5 = always.

23-To know, be shown, the route to a destination.

1 = never, 2 = sometimes, 3 = often, 4 = very often, 5 = always.

24-To look for an alternative route when, for example, encountering road congestion or construction work.

1 = never, 2 = sometimes, 3 = often, 4 = very often, 5 = always.

25-When one is lost or disoriented.

1 = never, 2 = sometimes, 3 = often, 4 = very often, 5 = always.

26-To know where you are.

1 = never, 2 = sometimes, 3 = often, 4 = very often, 5 = always.

27-To know where a destination is located.

1 = never, 2 = sometimes, 3 = often, 4 = very often, 5 = always.
